# Analysis of the etiology of infection and antimicrobial resistance in chronic critically ill patients: a longitudinal study

**DOI:** 10.62838/jccm-2026-0031

**Published:** 2026-10-30

**Authors:** Raquel Bergamasco e Paula, Marcos Toshiyuki Tanita, Claudia Maria Dantas de Maio Carrilho, Gilselena Kerbauy, Cintia Grion

**Affiliations:** Centro de Ciências da Saúde, Universidade Estadual de Londrina, Londrina, Brazil

**Keywords:** Critically ill patients, Healthcare-associated infections, Carbapenem resistance, Drug Resistance

## Abstract

**Background:**

Hospital acquired infections and sepsis play an important role both as a triggering factor for chronic critically illness [CCI] and as a common complication of this syndrome. The aim of this study is to describe the clinical progression of sepsis episodes in CCI patients in the intensive care unit [ICU] of a Brazilian public hospital.

**Materials and Methods:**

A retrospective longitudinal study was conducted in a general ICU, from July 2022 to June 2023. The convenience sample consisted of 779 patients. Demographic, clinical, and microbiological data were analyzed.

**Results:**

The incidence of CCI was 20.9% (n=166). Septic shock on the first day of hospitalization occurred in 60.2%, and the hospital mortality rate was 50%. A total of 362 episodes of infection were recorded. Ventilator-associated pneumonia [VAP], urinary tract infection (UTI), and bloodstream infection (BSI) were the most prevalent infections (40%, 20.7%, and 19.6%, respectively). Bacteria of the ESKAPE group - *(Enterococcus spp., Staphylococcus aureus, Klebsiella pneumoniae, Acinetobacter baumannii, Pseudomonas aeruginosa and Enterobacter spp.)* accounted for 57% of all positive samples, showing high bacterial resistance, with a predominance of resistance to carbapenems in Gram-negative bacilli.

**Conclusions:**

The incidence of CCI at the research institution was high. Factors such as SAPS3 score, multiple infections, and diagnosis of shock or sepsis were predictors of in-hospital mortality. VAP was the most prevalent site of the recorded infection episodes. Multidrug-resistant bacteria from the ESKAPE group were identified from admission, and formal temporal analysis demonstrated a statistically significant increase in antimicrobial resistance over the course of hospitalization, particularly when the main Gram-negative pathogens were analyzed together.

## Introduction

Technological advances in intensive care medicine have had an impact on increased patient survival, however, they have also led to the growth of a population marked by persistent organic dysfunctions and dependence on artificial life support [1,2], a condition known as chronic critical illness [CCI]. CCI represents approximately 11% of admissions to Intensive Care Units [ICU] [3], being responsible for high occupancy rates of ICU beds and costing billions of dollars per year in the United States [4,5]. CCI has a high mortality rate, reaching 62% in 1 year [6], and a high prevalence of limiting functional sequelae in those who survive [7].

Although there is still no unanimous definition of CCI in the literature, it is known that it is not just a temporal progression of the acute disease, but rather a complex syndrome resulting from a dysfunctional adaptive response, involving mechanisms of persistent inflammation, immunosuppression, and catabolism, as well as metabolic and hematopoietic alterations [8]. The reasons for this imbalance are multifactorial, with previous comorbidities and frailty [9] being highlighted, as well as external factors directly related to the care received, such as inappropriate use of antibiotics, mechanical ventilation [MV] with non-protective parameters, unrestricted fluid infusion, insufficient nutrition, excessive sedation, and increased risk of new infections due to invasive devices [10,11].

In this context, sepsis plays an important role both as a triggering factor for CCI and as a common complication of this syndrome [12,13]. In addition to being an isolated risk factor for mortality, approximately half of critically ill patients diagnosed with sepsis do not experience rapid recovery from the condition, remaining with some degree of organic impairment, which results in longer hospital and ICU stays [9,14]. This condition, associated with immunological exhaustion, exposure to multidrug-resistant [MDR] microorganisms, and numerous barrier breaches by invasive devices, favors new infections, perpetuating the condition and reducing survival [15,16]. Although global and national mortality attributed to sepsis and septic shock have reduced over the past few years, the incidence and complications have increased, reflecting both improvements in diagnostic tools and the available therapeutic arsenal and failure to fully reverse organ dysfunctions [17,18,19].

The complexity of these patients, combined with the unfavorable prognosis and the local and global impact of associated microbial resistance, demands adequate control and prevention plans. Knowing the trajectory of these patients and their relationship with the incidence of hospital acquired infections [HAI] could be useful in structuring such measures. Thus, the objective of the current study was to describe the clinical progression of chronic critically ill patients in the ICU of the University Hospital of Londrina, particularly considering the site of infection, etiological agent, and antimicrobial resistance profile in sepsis episodes.

## Methods

This study was approved by the local ethics committee under opinion number 6.159.589 and CAAE number 70588323.5.0000.5231. Signing of the informed consent form was waived.

This is a retrospective longitudinal study that analyzed all adult patients admitted to a general ICU with 10 beds, from July 2022 to June 2023, and who did not meet the exclusion criteria: readmissions to the ICU, transfer from another ICU, having prerogatives of limited therapeutic support or in palliative care (PC) upon admission to the ICU.

CCI was defined as an ICU stay of eight days or more, associated with at least one of the following conditions: MV for at least 96 hours, presence of tracheostomy [TCT], sepsis, intracranial hemorrhage, neurological patient, traumatic brain injury, post-neurosurgery, or coma [20]. HAIs were defined according to criteria established by the Brazilian Health Regulatory Agency – ANVISA, which establishes a count of minimum colony forming units (CFU) associated with the presence of specific symptoms to characterize infection. Thus, in ventilator-associated pneumonia (VAP), only isolated microorganisms with ≥10^5^ CFU in tracheal aspirate and ≥10^4^ in bronchoalveolar lavage (BAL) were considered representative of infection, and in urinary tract infections (UTIs), microorganisms with ≥10^5^ CFU. For the diagnosis of bloodstream infection (BSI), the presence of a positive blood culture plus the presence of specific symptoms was used, with S. epidermidis infections being considered only when this microorganism was isolated in more than one blood culture at different sites and times. Microorganisms isolated in surveillance cultures (swabs) were not considered for infection diagnosis. All infections treated in the ICU were reviewed and monitored by infectious disease specialists dedicated to hospital infection control [21]. Infection episodes evaluated in CCI patients were considered ICU-acquired infections if they presented as a new-onset infection occurring 48 hours or more after admission to the ICU. Sepsis diagnosis followed the third international consensus definitions for sepsis and septic shock recommendations [22].

Readmission was defined as any patient who had an indication for unscheduled readmission to the ICU after discharge during the same hospital stay. Chronic disease was defined according to the description of the Charlson comorbidity index [23].

General data collected for all ICU admissions were sex, age, type of admission, ICU admission diagnosis, length of hospital and ICU stay, and vital status at ICU discharge and hospital discharge.

The data collected during ICU admission were the presence of a chronic disease, need for MV, use of vasopressors, and need for hemodialysis [HD], as well as the scores for the Simplified Acute Physiology Score III [SAPS3] [24] and Sequential Organ Failure Assessment [SOFA] [25]. The data collected after discharge from the ICU were related to the presence of a new sepsis event and the need for readmission to the ICU during the same hospital admission.

During the entire hospital stay, biological samples were collected for culture and identification of the etiological agent and its respective sensitivity profile or antimicrobial resistance analyses; each microorganism isolated from an infection episode was counted as an individual isolate. When more than one microorganism was reported in the same episode, each pathogen was counted separately. Resistance rates were calculated using the number of isolates of each microorganism with available antimicrobial susceptibility data as the denominator. Empirical treatments, missing microbiological data, and isolates without a defined susceptibility profile were excluded from the denominator for resistance-rate calculations.

The identification of bacterial isolates was performed using standardized methodologies [26] with both conventional and automated techniques, in the Vitek^®^ 2 system (bioMeriéux-Marcy-l'Étoile, France). Sensitivity tests were performed according to the technique recommended by the manufacturer of the automated system and/or using the disk diffusion method (OX-OID, Basingstoke, Hants, UK), both based on standardization, clinical cutoff points, and interpretation according to criteria established by regulations of the Brazilian Committee on Antimicrobial Susceptibility Testing [27]. Resistance to polymyxins was confirmed by manual Broth Microdilution.

The dependent variable analyzed was hospital mortality. The independent variables were risk factors, including clinical and demographic variables, SAPS3 severity score, and SOFA organ dysfunction score.

The sources used for data collection were the patient's electronic medical record and the Epimed Monitor ICU Database^®^ platform [28]. Patients were followed until hospital discharge, with the outcome considered as survival or non-survival.

### Statistical analysis

Continuous variables are expressed as mean and standard deviation or as median and quartiles. Categorical variables are expressed as absolute and relative frequency. Data are presented in figures and tables. The Student's t-test, or non-parametric equivalent (Mann-Whitney) was used to compare continuous variables. Categorical variables were compared using Pearson's Chi-square test. Bivariate analysis and logistic regression were performed to analyze variables predicting the outcome. Multicollinearity among candidate predictors for the multivariable logistic regression model was assessed using the variance inflation factor (VIF), obtained from an auxiliary multiple regression model including the same independent variables. A VIF greater than 5 was considered indicative of relevant multicollinearity. Model performance was assessed by discrimination and calibration. Discrimination was evaluated using the area under the receiver operating characteristic curve. Calibration was assessed using the Hosmer-Lemeshow goodness-of-fit test, calibration intercept, calibration slope, and comparison of observed and predicted hospital mortality across quartiles of predicted risk. Calibration intercept and calibration slope were estimated using the linear predictor from the final logistic regression model. Because calibration was assessed in the same sample used to develop the model, these estimates should be interpreted as apparent calibration. Missing data were handled by complete-case analysis. In MedCalc, empty cells, user-defined missing values, and values causing computational errors are excluded from the statistical analyses. Therefore, patients were included in the multivariable logistic regression model only when data were available for the outcome and for all predictors included in the model. No imputation procedures were performed.

For the temporal analysis of antimicrobial resistance, isolates were grouped according to the time from ICU admission to infection diagnosis: days 1–7, days 8–14, days 15–21, and ≥22 days. Resistance rates were calculated using the number of isolates with available antimicrobial susceptibility data in each time interval as the denominator. Isolates were classified as resistant when carbapenem resistance, polymyxin resistance, ESBL production, pan-resistance, or vancomycin resistance was reported, according to the pathogen analyzed. Susceptible isolates were classified as non-resistant. A Cochran-Armitage test for trend was used to evaluate changes in resistance rates across hospitalization periods. As a complementary analysis, logistic regression was performed using resistance status as the dependent variable and time from ICU admission as the independent variable, with adjustment for pathogen type.

The significance level used was 5% and the analyses were performed using the software MedCalc for Windows, version 9.3.2.0 (MedCalc Software, Mariakerke, Belgium).

## Results

During the study period, a total of 792 patients over 18 years of age were admitted to the ICU, of whom 13 (1.6%) were excluded due to the exclusion criteria, resulting in 779 patients. Of these, 166 (21.3%) met the criteria adopted for CCI (Figure 1).

**Fig. 1.**
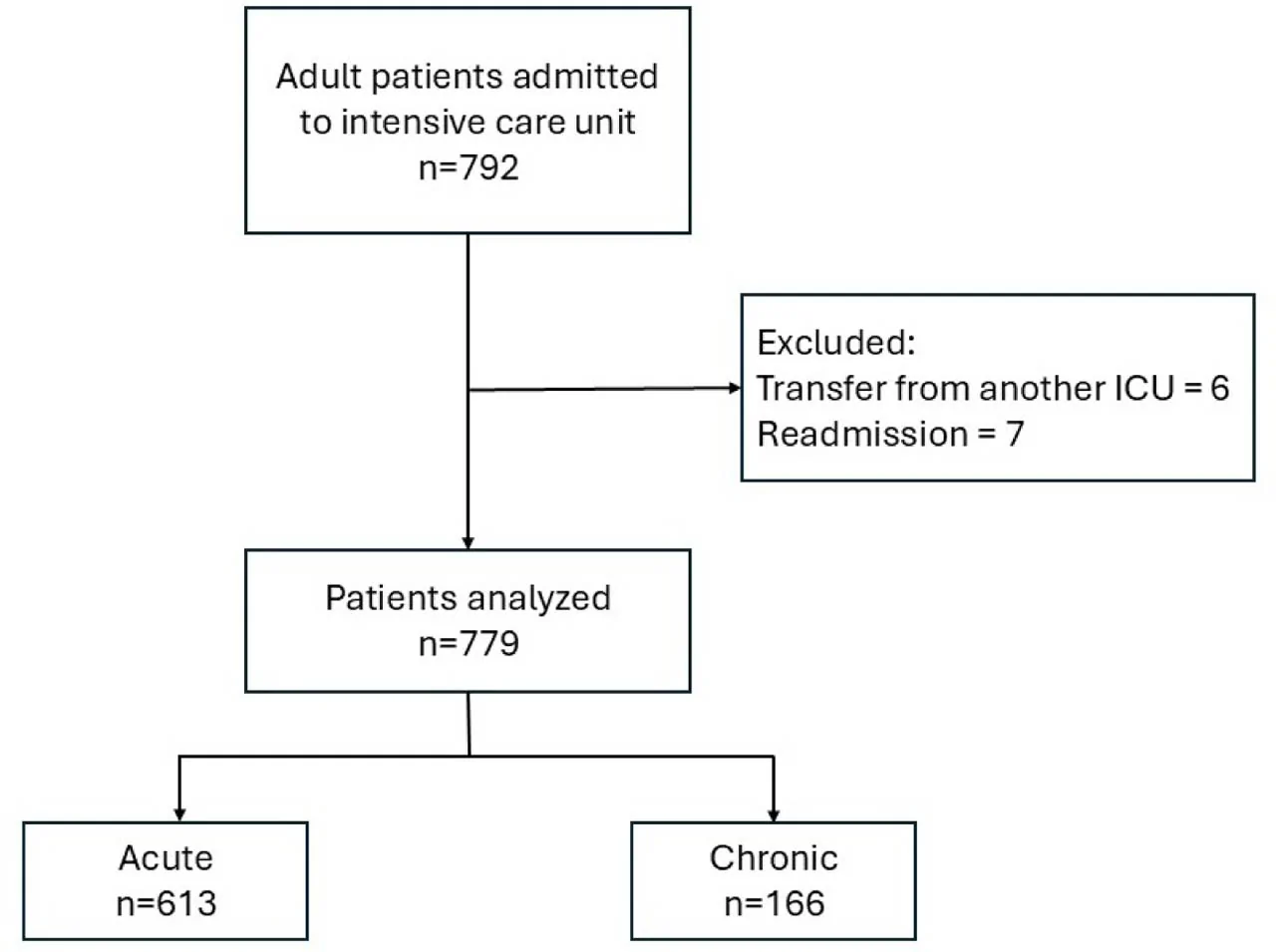
Flowchart of patients analyzed in the study

Chronic critical patients were predominantly men (60.8%), with a median age of 60 (45–73) years, a Charlson comorbidity index of 1, and the presence of sepsis on ICU admission in 23.5% of cases. When compared to short-stay patients, patients with CCI had higher median SAPS3 and SOFA severity scores (64.82 and 9, respectively). Likewise, the presence of septic shock on the first day of hospitalization (60.2%), and mortality rate in the ICU (33.7%) and hospital (50%) were also higher. Readmission to the ICU was observed in 4.2% of chronic critically ill patients. As expected for this group, both the median length of hospital stay (26 days) and the median length of MV (12 days) were higher, as was the need for TCT (42.2%) and HD (39.8%), and the use of vasoactive drugs (77.7%) (Table 1). Regarding the indication of PC, 12% of patients with CCI received this type of therapeutic plan at some point during hospitalization, after admission to the ICU.

**Table 1:** Demographic and clinical characteristics of patients admitted to the Intensive Care Unit

	**CCI (n=166)**	**Non-CCI (n=613)**	**p value**
Age (years)	60 (45–73)	65 (51–74)	0.02^*^
Male Sex (%)	101 (60.8)	344 (56.1)	0.27^†^
ICU indication			
Medical (%)	106 (63.9)	190 (31)	
Elective Surgery (%)	13 (7.6)	201 (32.8)	<0.0001^‡^
Surgical Emergency (%)	47 (28.3)	222(36.2)	
SAPS3	64.8 (51–77)	45 (35–56)	<0.0001^‡^
Admission SOFA	9 (6–11)	3 (1–6)	<0.0001^‡^
Sepsis D1 (%)	39 (23.5)	104 (17)	0.05^†^
Shock D1 (%)	100 (60.2)	109 (17.8)	<0.0001
ICU Sepsis (%)	90 (54.2)	136 (22.2)	<0.0001
ICU Shock (%)	129 (77.7)	210 (34.3)	<0.0001
ICU mortality (%)	56 (33.7)	71 (11.6)	<0.0001
Indication for readmission (%)	7 (4.2)	15 (2.4)	0.22^†^
Sepsis/Post-ICU Shock (%)	18 (10.8)	15 (2.4)	<0.0001
Length of stay in the ICU	14 (10–22)	2 (1–3)	<0.0001
Length of hospital stay	26 (17–43)	8 (4–14)	<0.0001
Hospital mortality (%)	83 (50)	109 (17.8)	<0.0001
Palliative Care (%)	20 (12)	4 (0.6)	<0.0001
MV time	12 (8.5–22)	1 (1–4)	<0.0001
TCT (%)	70 (42.2)	17 (2.8)	<0.0001
VAD (%)	129 (77.7)	174 (28.4)	<0.0001
HD (%)	66 (39.8)	44 (7.2)	<0.0001
HD time	6.5 (3–10)	2 (1–3)	<0.0001

Legend: CCI – chronic critical illness; ICU – Intensive Care Unit; SAPS 3 – Simplified Acute Physiologic Score; SOFA – Sequential Organ Failure Assessment; MV – mechanical ventilation; TCT – Tracheostomy; VAD – Vasoactive Drug; HD – Hemodialysis

* Mann Whitney test;

† Fisher's exact test;

‡ Student t test.

Bivariate analysis was performed between the independent variables and hospital mortality. The variables were included in the logistic regression model using the stepwise forward method to the same outcome. SAPS3, presence of septic shock during and after ICU admission, and diagnosis of more than one HAI were independent predictors of hospital mortality (Table 2). No relevant multicollinearity was detected among the predictors included in the final multivariable model, as all VIF values were below 5. The final logistic regression model showed good apparent discrimination, with an area under the ROC curve of 0.818. Apparent calibration was acceptable, with a calibration intercept close to zero and a calibration slope of 1.00. Observed and predicted mortality were also compared across quartiles of predicted risk. Observed mortality values were 16.7%, 31.7%, 70.7%, and 81.0% from the lowest to the highest risk quartile, while the corresponding predicted mortality values were 14.8%, 42.0%, 58.9%, and 84.4%, respectively.

**Table 2:** Bivariate analysis and logistic regression for the outcome of hospital mortality in patients with chronic critical illness

**Bivariate analysis**				**Logistic regression**			

Variable	Odds ratio	95% CI	P	Variable	Odds ratio	95% CI	p
Shock in the ICU	7.65	2.98 to 19.63	<0.0001	Shock in the ICU	5.55	1.9 to 16.21	0.0017
Sepsis/Post-ICU Shock	5.88	1.63 to 21.18	0.0067	Sepsis/Post-ICU Shock	4.75	1.07 to 21.09	0.0404
Number of infections	1.50	1.24 to 1.81	<0.0001	Number of infections	1.38	1.13 to 1.69	0.0019
SAPS3	1.04	1.02 to 1.06	0.0001	SAPS3	1.03	1.01 to 1.05	0.0134
SOFA	1.15	1.05 to 1.25	0.0017				
Shock on ICU admission	2.52	1.33 to 4.78	0.0047				
Charlson	1.27	1.01 to 1.60	0.0401				

Hosmer & Lemeshow Test: P=0.823

ROC curve analysis: AUC=0.818.

A total of 362 episodes of infection were diagnosed in 166 patients with CCI. VAP accounted for 145 (40%) episodes, followed by 75 cases of UTI (20.7%) and 71 cases of BSI (19.6%). Other sites with less representation were surgical site (17; 4.7%), pneumonia not associated with MV (16; 4.4%), skin and soft tissue (10; 2.8%), gastrointestinal tract (9; 2.5%), bone (6; 1.6%), abdomen (4; 1.1%), central nervous system (4; 1.1%), and an undetermined focus (5; 1.4%). Thirty-six patients (21.7%) did not present infection during hospitalization, 73 patients (44%) presented 1 to 2 episodes, 43 (25.9%) had 3 to 5 episodes, and 14 (8.4%) presented 6 or more episodes of infection. When analyzing the impact of this sum on the mortality outcome, a progressive increase was observed, with a minimum chance of survival from the eighth episode of infection (Figure 2).

**Fig. 2.**
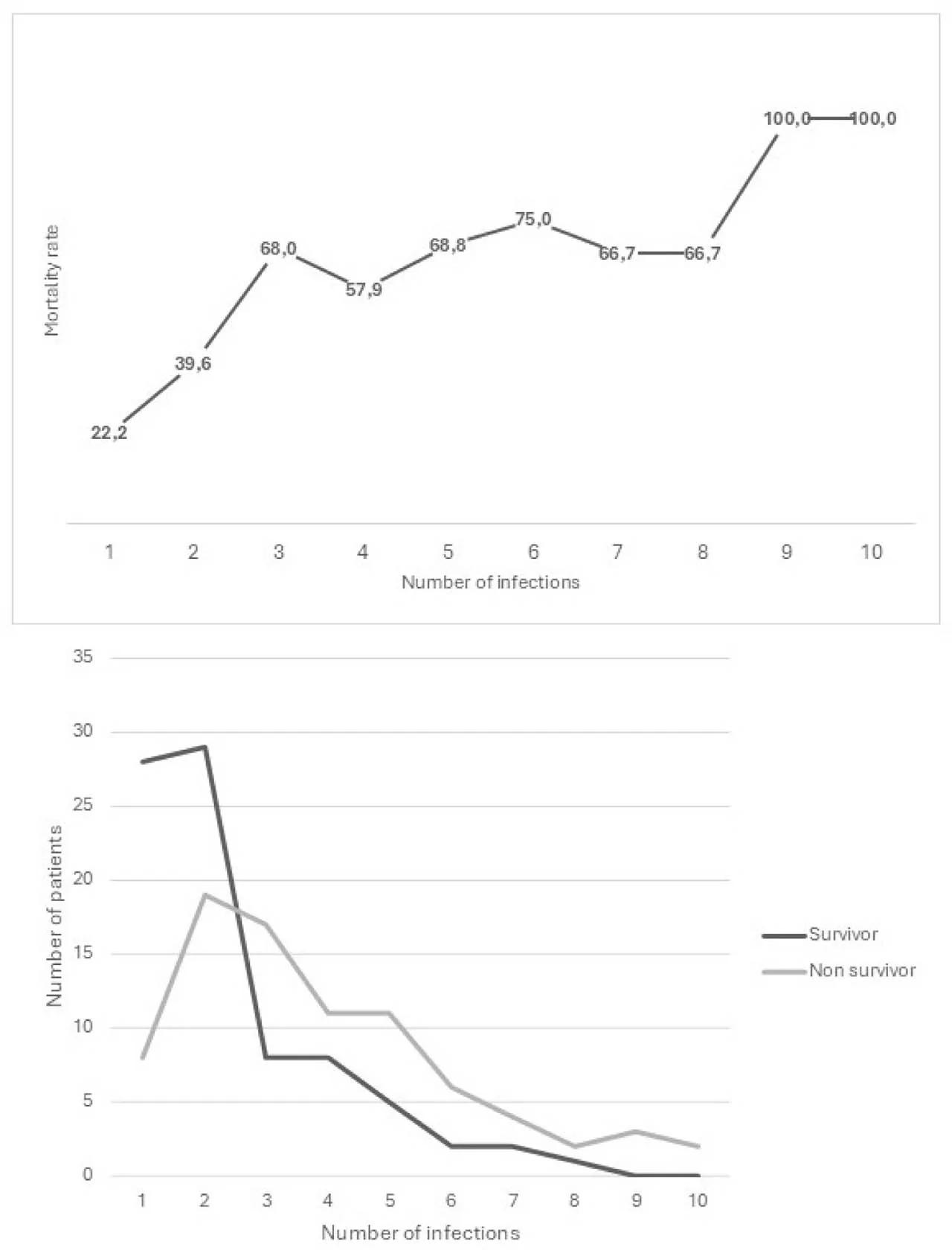
Mortality rate and distribution of the patients in relation to the number of infections Legend: Each infection episode was considered as a new one if it occurred on a new site of infection or if a new microorganism was isolated.

A total of 393 positive cultures for microorganisms were found in the infection episodes. Non-Glucose Fermenting Gram-Negative Bacilli [NFGNB] and *Enterobacteriaceae* were the predominant infectious agents (30.9% and 29.3% respectively), followed in smaller numbers by *Enterococcus spp.* (9.4%), *Candida spp.* (8%), Coagulase Negative *Staphylococci* (5.8%), *Staphylococcus aureus* (4%), and *Clostridioides* (2.3%), among others. Cultures with negative results represented 8% of the samples collected. In the tracheal secretion aspirate, 51 (29.1%) samples were positive for *Acinetobacter baumannii*, 34 (19.4%) for *Klebsiella pneumoniae*, 29 (16.6%) for *Pseudomonas aeruginosa*, and 12 (6.8%) for *Staphylococcus aureus*. In urine samples, 21 (25.6%) cases of *Klebsiella pneumoniae* were isolated, 12 (14.6%) of *Candida albicans*, 8 (9.7%) of *Candida glabrata*, and 6 (7.3%) of *Pseudomonas aeruginosa*. In blood cultures, 14 (18.2%) cases of *Klebsiella pneumoniae* were isolated, 8 (10.4%) of *Staphylococcus epidermidis*, 6 (7.8%) of *Enterococcus faecalis*, 5 (6.5%) of *Enterococcus faecium*, and 5 (6.5%) of *Acinetobacter baumannii*.

Resistance rates were calculated using as the denominator the number of isolates of each microorganism with available antimicrobial susceptibility data as. As observed in Figure 3, among 81 *Klebsiella pneumoniae* isolates, 47/81 (58.0%) were carbapenem-resistant, 18/81 (22.2%) were polymyxin-resistant, 4/81 (4.9%) were ESBL-producing, 1/81 (1.2%) was pan-resistant, and 11/81 (13.6%) were susceptible. Among 69 *Acinetobacter baumannii* isolates, 61/69 (88.4%) were carbapenem-resistant, 7/69 (10.1%) were polymyxin-resistant, and 1/69 (1.4%) was susceptible. Among 47 *Pseudomonas aeruginosa* isolates, 26/47 (55.3%) were carbapenem-resistant, 1/47 (2.1%) was a possible ESBL-producing phenotype, and 20/47 (42.6%) were susceptible. Among 38 *Enterococcus spp*. isolates with available susceptibility profile, 17/38 (44.7%) were vancomycin-resistant and 21/38 (55.3%) were susceptible.

**Fig. 3.**
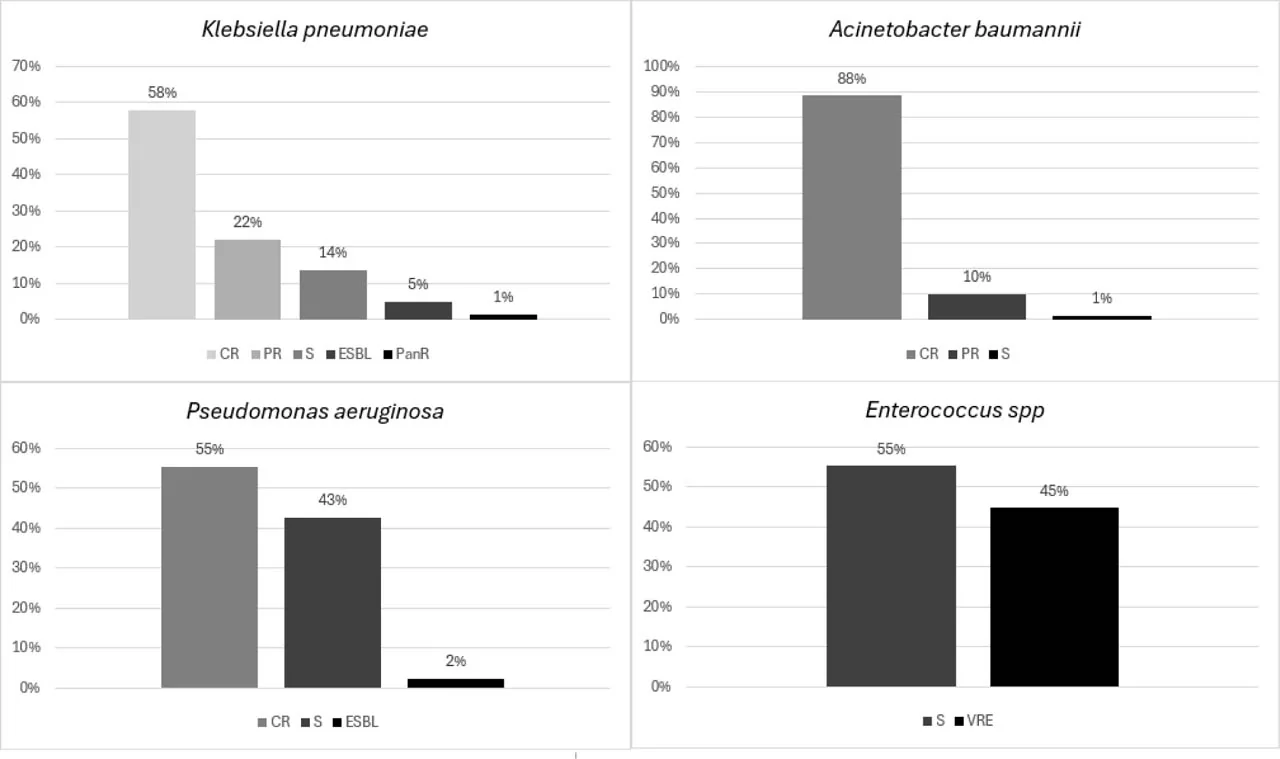
Frequency of antimicrobial resistance profiles of the main isolated pathogens Legend: Percentages were calculated using the number of isolates of each microorganism with available antimicrobial susceptibility data as the denominator: *Klebsiella pneumoniae* (n = 81), *Acinetobacter baumannii* (n = 69), *Pseudomonas aeruginosa* (n = 47), and *Enterococcus spp*. (n = 38). When more than one microorganism was isolated in the same infection episode, each microorganism was counted as an individual isolate. ESBL: extended-spectrum beta-lactamase producer; CR: carbapenem-resistant; PR: polymyxin-resistant; Pan-R: resistant to all antimicrobials tested; VRE: vancomycin-resistant *Enterococcus*; S: susceptible.

A statistically significant increase in antimicrobial resistance was observed over the course of hospitalization (Figure 4). Among the main pathogens analyzed together, resistance increased from 28/48 isolates (58.3%) in the first week to 82/96 isolates (85.4%) after 21 days of hospitalization (χ^2^ for trend = 12.06; p = 0.00052). When the analysis was restricted to the main Gram-negative pathogens, resistance increased from 26/38 isolates (68.4%) in the first week to 73/82 isolates (89.0%) after 21 days (χ^2^ for trend = 6.44; p = 0.011). In pathogen-adjusted logistic regression, each additional day from ICU admission was associated with increased odds of resistance among the main Gram-negative pathogens (OR = 1.047; 95% CI, 1.014–1.082; p = 0.0047) (Table III).

**Fig. 4.**
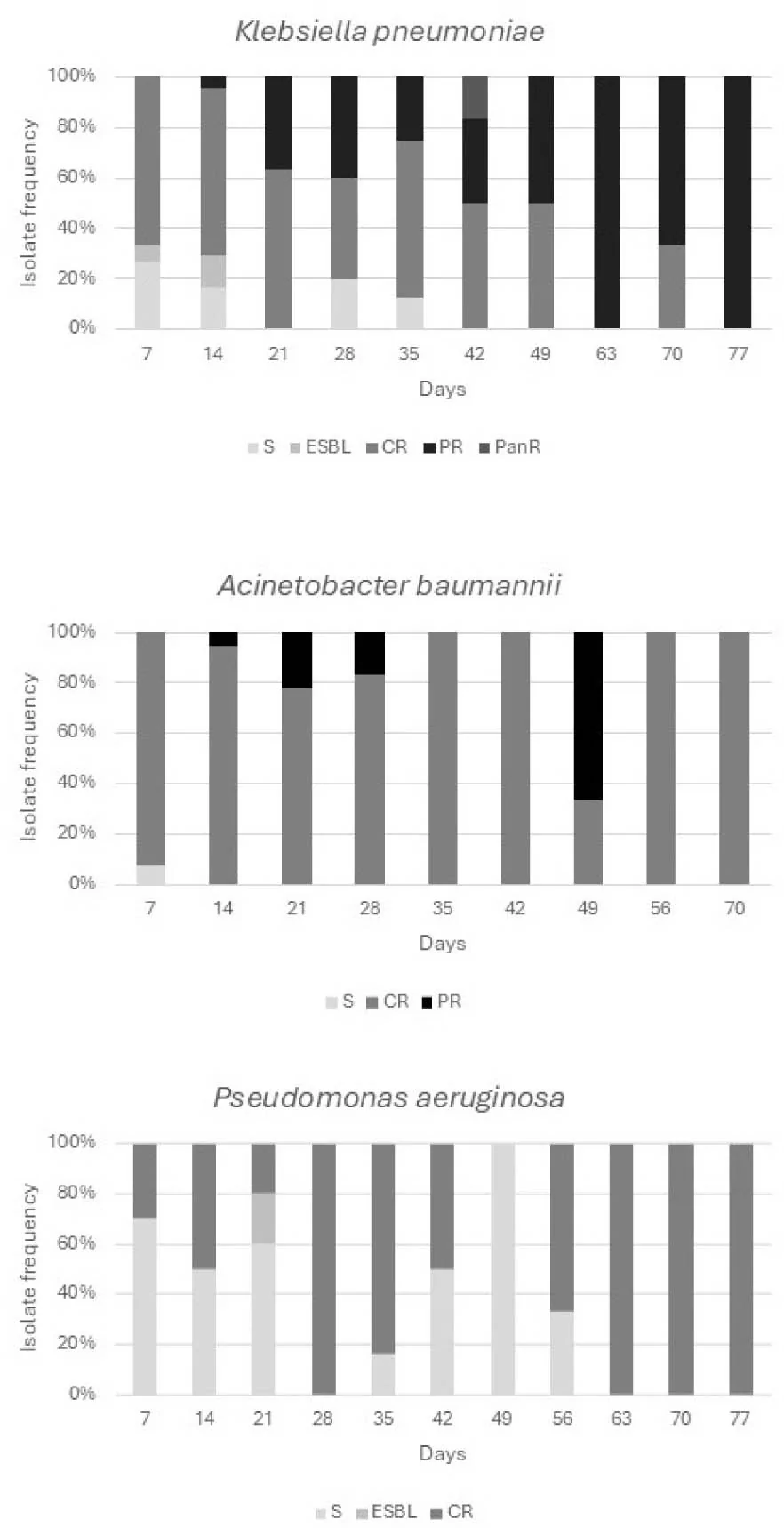
Graphs showing the evolution of the antimicrobial sensitivity profile of the main microorganisms throughout the hospitalization period Legend: S = sensitive; ESBL = extended-spectrum beta-lactamase producer; CR = carbapenemase producer; PR = polymyxin resistance; PanR = resistance to all antimicrobials tested.

**Table 3:** Temporal trend in antimicrobial resistance according to hospitalization period.

**Group**	**Days 1–7 n/N (%)**	**Days 8–14 n/N (%)**	**Days 15–21 n/N (%)**	**≥22 days n/N (%)**	**χ^2^ for trend**	**p for trend**
Main pathogens	28/48 (58.3%)	46/60 (76.7%)	26/31 (83.9%)	82/96 (85.4%)	12.06	0.00052
Main Gram-negative pathogens	26/38 (68.4%)	44/52 (84.6%)	22/25 (88.0%)	73/82 (89.0%)	6.44	0.011
K. pneumoniae	11/15 (73.3%)	20/24 (83.3%)	11/11 (100%)	28/31 (90.3%)	—	0.100
A. baumannii	12/13 (92.3%)	20/20 (100%)	9/9 (100%)	27/27 (100%)	—	0.136
P. aeruginosa	3/10 (30.0%)	4/8 (50.0%)	2/5 (40.0%)	18/24 (75.0%)	—	0.014
Enterococcus spp.	2/10 (20.0%)	2/8 (25.0%)	4/6 (66.7%)	9/14 (64.3%)	—	0.013

<xsref0>Resistance rates are presented as n/N (%), where N represents the number of isolates with available antimicrobial susceptibility data in each hospitalization period. Main pathogens included *Klebsiella pneumoniae, Acinetobacter baumannii, Pseudomonas aeruginosa*, and *Enterococcus spp*. Main Gram-negative pathogens included *Klebsiella pneumoniae, Acinetobacter baumannii*, and *Pseudomonas aeruginosa*. Resistance was defined as carbapenem resistance, polymyxin resistance, ESBL production, pan-resistance, or vancomycin resistance, according to the pathogen analyzed.

## Discussion

This single-center study described the clinical progression of patients with CCI admitted to a general ICU, focusing on episodes of HAIs, and the most prevalent sites and microorganisms involved. In addition, survival, readmission rates, and PC indications in these patients throughout their hospital stay were evaluated. The patients analyzed presented with high severity and were prone to complications and organ dysfunctions, with multiple episodes of HAIs, often caused by multi-drug-resistant microorganisms. The pattern of antimicrobial resistance intensified with the patients' illness chronicity.

Although the definition of CCI lacks uniformity among different research groups, hindering comparisons of the results obtained, our study is in accordance with the literature, which presents incidences of 5–33% [12,13,29]. It is worth noting that the development of the study in a general ICU of a university hospital may have contributed to the high number of cases found [11].

As expected, individuals who developed CCI presented, upon admission to the ICU, high severity and prognosis scores and significant organ impairment [3]. Loss et al., reported that age and sex were not determinants for the presence of CCI [14]. One possible explanation is that the group of acute patients was made up of both rapid responders and patients who died in the first few days, compromising this comparison. The mortality rate observed during the period of hospitalization agrees with data published by other groups of researchers [3], although most studies carrying out this analysis cover a period of one year, and obtain even higher values, of between 40–68% [10,29,30].

The distribution of the infections analyzed, considering only those diagnosed from the second day of hospitalization and, therefore, associated with health care, followed a pattern like that previously described by other authors [31,32]. VAP was the most prevalent of the recorded HAIs, followed by UTI and BSI. The occurrence of these infections may be related to the degree of complexity and dependence of patients on artificial life support, time of exposure to invasive devices, adherence by health professionals to sets of preventive measures for hospital infections, and virulence and antimicrobial resistance of the pathogens involved [33].

Almost 80% of the patients with CCI had multiple episodes of infection. For VAP cases, the peak occurred in the second week of hospitalization, decreasing after this period, and coinciding with the median time of MV in this group. In absolute terms, the number of infections showed a progressive decline, explained by the reduced number of survivors analyzed over time. The greater susceptibility to infections and recurrence of cases were observed in many previously published studies, which sought to explain these findings through pathophysiological changes, such as immune exhaustion and a persistent inflammatory state, among other causes [9,34,35].

The main pathogens isolated in the current research were *Klebsiella pneumoniae*, *Acinetobacter baumannii,* and *Pseudomonas aeruginosa*, which were, together, responsible for almost half of the positive cultures. The phenomenon observed in this study, with a reduction in the sensitivity profile of these microorganisms, can be explained by their great and rapid capacity to develop adaptation and survival mechanisms, through selection pressure, mutations, and transfer of genetic material vertically and horizontally [36]. These mechanisms, whether natural, induced, or acquired, give this group the title of critical and priority microorganisms by the World Health Organization regarding global public health, due to their relationship with high mortality rates and consumption of resources in health institutions. The presence of bacteria from the ESKAPE group, although common to most highly complex intensive care services, indicates the severity of these infections and calls for the need to intensify hospital infection control measures through antimicrobial stewardships [37,38]. Likewise, the occurrence of sensitive strains in the 3 subgroups analyzed, although not as significant, reinforces the need to adopt the rational use of these medications, in which “less is often more”.

HAIs are thus configured as the connecting point between two worrying scenarios within the hospital organization. On the one hand, there is the inability of patients with CCI to sustain satisfactory clinical improvements, and on the other, there is the continuous selection pressure on pathogenic agents, inducing them to adapt through resistance mechanisms. Strategies such as intensifying infection prevention bundles, antimicrobial stewardship programs, continuing education programs, implementing tools for early and systematic microbiological diagnosis through the incorporation of new technologies, such as molecular biology and rapid testing for carbapenemases detection, the increased presence of the infectious disease specialists from the emergency department in the ICU, and even a palliative approach in cases where repeated therapeutic failures are observed could be of great value in changing this paradigm.

There are limitations to be considered in this study. Because it was carried out in a single center, extrapolation of the findings to populations with similar characteristics should be performed with caution. The retrospective design and convenience sampling introduce selection bias and unmeasured confounding. Using an 8-day instead of a 21-day cutoff may have captured "prolonged acute" patients, so interpretation of the results should consider the definition adopted in the present study. The strengths of this study include the fact that it covered twelve consecutive months, to avoid seasonal bias, and it is one of the few studies carried out in Latin America that describes the main HAIs in CCI, highlighting the microorganisms involved and antimicrobial resistance profile over time.

## Conclusion

The incidence of CCI at the research institution was high. Factors such as SAPS3 score, multiple infections, and diagnosis of shock or sepsis were predictors of in-hospital mortality. VAP was the most prevalent site of the recorded infection episodes. Multidrug-resistant bacteria from the ESKAPE group were identified from admission, and formal temporal analysis demonstrated a statistically significant increase in antimicrobial resistance over the course of hospitalization, particularly when the main Gram-negative pathogens were analyzed together.
